# Translation and adaptation of an international questionnaire to measure usage of complementary and alternative medicine (I-CAM-G)

**DOI:** 10.1186/1472-6882-12-259

**Published:** 2012-12-20

**Authors:** Meike Lo Re, Stefan Schmidt, Corina Güthlin

**Affiliations:** 1 Institute of General Practice, Goethe University Frankfurt, Frankfurt am Main, Germany; 2 Department Psychosomatic Medicine, University Medical Center Freiburg, Freiburg, Germany; 3 Institute for Transcultural Health Studies, European University Viadrina, Frankfurt (Oder), Germany

## Abstract

**Background:**

The growing body of data on prevalence of complementary and alternative medicine (CAM) usage means there is a need to standardize measurement on an international level. An international team has published a questionnaire (I-CAM-Q), but no validation has yet been provided. The aim of the present study was to provide a German measurement instrument for CAM usage (I-CAM-G) which closely resembles the original English version, and to assess it’s performance in two potential samples for measuring CAM usage.

**Methods:**

The English I-CAM-Q questionnaire was translated into German, and adapted slightly. The resulting I-CAM-G questionnaire was then pre-tested on 16 healthy volunteers, and 12 cognitive interviews were carried out. The questionnaire was employed in a sample of breast cancer patients (N = 92, paper and pencil), and a sample from the general population (N = 210, internet survey). Descriptive analyses of items and missing data, as well as results from the cognitive interviews, are presented in this paper.

**Results:**

The translated questionnaire had to be adapted to be consistent with the German health care system. All items were comprehensible, whereby some items were unambiguous (e.g. CAM use yes/no, helpfulness), while others gave rise to ambiguous answers (e.g. reasons for CAM use), or high rates of missing data (e.g. number of times the CAM modality had been used during the last 3 months). 78% of the breast cancer patients and up to 85% of a sample of the general population had used some form of CAM.

**Conclusions:**

Following methodologically sound and comprehensive translation, adaptation and assessment processes using recognized translation procedures, cognitive interviews, and studying the performance of the questionnaire in two samples, we arrived at a German questionnaire for measuring CAM use which is comparable with the international (English) version. The questionnaire appropriately measures CAM use, with some items being more appropriate than others. We recommend the development of a short version.

## Background

Although studies show that interest in complementary and alternative medicine (CAM) is growing throughout the world [1-3], it is still difficult to find reliable data on the frequency of CAM use in, for example, specific diagnosis-related groups. This is partly because the reasons for using CAM vary considerably. Patients may use CAM as an alternative, i.e. instead of conventional therapy, or complementarily, and thus in addition to conventional treatments. The latter is often the case in oncology, and it is from this field that most published data is available [4-7]. However, even in this well studied area, pooled data shows the frequency of CAM use to range from 25% to 50% within a given patient population, with the percentage being highly dependent on region and the measurement time frame [8]. By studying this data, the most significant reasons for data variations become evident: Firstly, a fixed definition of CAM is necessary, as some surveys include praying or relaxation techniques, for example [9,10], while others do not [1]. Depending on the definition of CAM, empirical data shows that CAM usage may range from 11% to 72% in the same patient group [11].

Secondly, there is no standardized methodology for measuring CAM usage. Surveys may ask about lifetime usage [12], or solely about usage during the trajectory of a specific disease [4].

A systematic review of survey data on CAM usage in cancer patients shows that - apart from the measurement time frame - rates of CAM usage are also largely dependent on whether the participants were interviewed face-to-face or by means of a paper-pencil questionnaire, whether respondents were prompted by means of a list of CAM therapies, and whether surveys restricted CAM usage to particular CAM modalities [8].

As a result of a complete lack of measurement standardization for CAM usage, the National Research Center in Complementary and Alternative Medicine (NAFKAM) in Norway carried out a workshop with the aim of developing a standardized questionnaire for measuring international CAM usage. Participants represented different countries (United States, Canada, Great Britain, Australia, Norway, Germany, Sweden and Denmark), came from a wide range of backgrounds (anthropology, sociology, nursing, health services, medicine, public health and pharmacy) and were specialists in different fields (survey design, cross-cultural research), and thus were able to develop a measurement instrument which covered both the most prominent types of CAM, as well as application methods in different countries, the I-CAM-Q [13].

In order to improve data comparability, the next step was to translate the international questionnaire for use in different countries and languages, and to assess it’s performance.

The aim of the present study was to provide a German measurement instrument for CAM usage (I-CAM-G) which closely resembles the original English version, and to assess it’s performance in two potential samples for measuring CAM usage.

As this is the first attempt to adapt the international questionnaire for use in a non-English-speaking country, experiences gathered here may help others to develop different language versions.

## Methods

### The original I-CAM-Q Questionnaire

The I-CAM-Q contains four sections. Section 1 (on page 1) asks about “Visiting health care providers”, section 2 (on page 2) about “Complementary treatments received from physicians (MDs)”, section 3 (on page 3) about the “Use of herbal medicine and dietary supplements” and section 4 (on page 4) about “Self-help practices”. The treatment modalities are presented in the form of a list, and respondents have to provide information on their usage over the previous 12 months (yes/no) and give details on the number of times the practitioner was seen/the treatment was received over the previous 3 months (except on page 3 where usage of herbal medicine and dietary supplements are asked about: respondents have to tick the remedies they are currently using). Respondents are also asked to indicate whether the CAM therapy was used on account of an acute illness/condition, a long-term illness, to improve general well-being, or for other reasons (if necessary). Finally, respondents are asked to indicate how helpful the CAM treatment had been. Please see the publication of the original version for the entire questionnaire [13] and Table 1 for the items.

**Table 1:** Questionnaire items

**Page**	**Items (incl. explanation given in questionnaire)**
1. Visiting health care providers	Homeopath (physicians who predominantly treat using homeopathy)
	Acupuncturist (physicians who provide acupuncture)
	Medical CAM specialist (physicians that provide a range of different CAM-therapies)
	Non-medical CAM specialist (Non-physicians that provide a range of different CAM-therapies)
	Osteopath (Physicians and non-physicians that provide osteopathy)
	Chirotherapist (Physicians and non-physicians that provide chirotherapy)
	Other Physicians and providers who treated you using CAM (please specify which treatment/therapy you received):
2. Complementary therapies received from physicians (MDs)	Homeopathy
	Acupuncture
	Herbal Medicine (tablets, pills, drops, ointments, teas, etc.)
	Manual Therapy
	Traditional Chinese Medicine
	Other CAM-therapies received from physicians (please specify the treatment/therapy)
3. Use of herbal medicines and dietary supplements	Homeopathic remedies (please specify the product)
	Herbs/Herbal remedies (please specify the product)
	Vitamins/Minerals (please specify the product)
	Other CAM products (please specify the product)
4. Self-help practices	Meditation
	Yoga
	Qigong
	Tai Chi
	Relaxation techniques
	Visualization
	Praying for own health
	Arts therapy
	Others (please specify the technique)

### The I-CAM-G questionnaire

The I-CAM-Q was translated and adapted for use in Germany. As the English version provides no information on the objectives of the questionnaire and gives no details on how to fill it in, we added instructions on the first page of the I-CAM-G. After pre-testing the German version and carrying out cognitive interviews, we then adapted the questionnaire slightly. The final version (please seeaddendum for the German questionnaire) was employed in a breast cancer sample using the paper-pencil-format, and on a sample of the general population using an internet questionnaire ( http://www.sphinxonline.net/lo_re/FREI-CAM/).

### Translation

The questionnaire was translated from English to German according to published guidelines for the translation of measurement instruments [14,15] using forward and backward translations by specialists in CAM research and native speakers of English and German.

After translating the questionnaire we prepared instructions on how to fill in the questionnaire, which we put on the first page of the I-CAM-G.

### Pretest

We pre-tested the questionnaire on 16 healthy volunteers to figure out how long it takes to fill out the questionnaire. We also made note of every question that was asked while the questionnaire was being filled in, and checked general comprehensibility. After the pre-tests we adapted in particular the format of the questionnaire. As the respondents had difficulties filling in the number of times we decided to offer two different versions of the page during the cognitive interviews. The new version asked about usage “daily, weekly, monthly, less than once a month” whereas the original version asked about the “Number of times you have used this practice in the last 3 months”.

### Cognitive interviews

After the preliminary pre-test we carried out cognitive interviews with 12 interviewees in order to gain detailed knowledge of the cognitive processes and potential pitfalls encountered when filling in the I-CAM-G. Cognitive interviews were recorded after obtaining consent from the participants. Cognitive interviews use different techniques such as “thinking aloud” and probing [16] to elicit information on cognitive processes and sources of response errors when responding to a questionnaire, and have been used successfully to test sources of misinterpretation in questionnaires for measuring CAM use [17].

**Figure 1:**
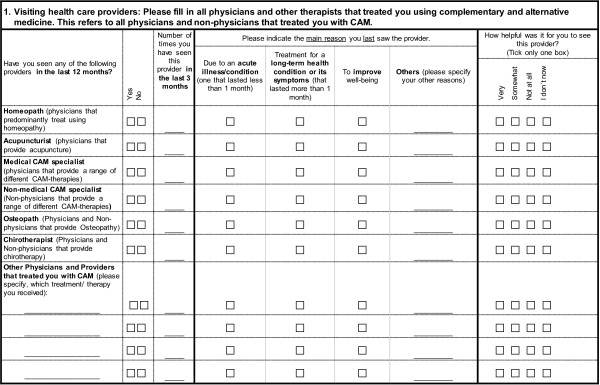
First page of I-CAM-G (German version, but translated to English).

The interviewees were patients of a practitioner of family medicine who either said they were acquainted with CAM, or that they did not use CAM at all, and were volunteering for being interviewed. After cognitive interviews we prepared a final version of the questionnaire in paper-pencil-format (see Table 1 and Figure 1) and an electronic version.

### CAM use in two samples

Collecting data with the I-CAM-G questionnaire comprised two steps:

1) Collecting data on CAM usage in a breast cancer sample, as this is the patient group for which the most data is available (e.g. [4-7]). In April 2010 we successively recruited patients from the breast cancer center of Goethe University hospital who gave consent to fill in the questionnaire in paper-pencil format. We tried to ensure to keep the sample as comprehensive as possible by only excluding patients on the grounds of not having a diagnosis of breast cancer.

2) Collecting data on the population level. Between August and October 2010 we prepared an electronic version for distribution via the internet. We used snow-ball sampling by initially sending out the questionnaire to e-mail addresses from the address-books of the authors CG, MLR, and SS, and asking recipients to fill in the questionnaire and to then forward the link to others.

In order to be able to split groups into frequent and infrequent users of CAM we asked the following introductory question: “What is your preferred choice when you are ill or feeling unwell?”, and provided the options to tick: “CAM”, “conventional medicine” or “I don‘t know”.

We also present some measurement properties like missing data and validity of herbal and homeopathic remedies here.

This research was carried out in full accordance with the Declaration of Helsinki and was approved by the IRB of the University of Frankfurt/Main, no reference number was assigned as no intervention was carried out.

### Data analysis and statistics

The data were stored in Microsoft Excel Spreadsheets and imported into IBM SPSS Statistics 19.0 for analysis. The analysis of performance measures was descriptive, and we collected data from several groups in order to compare groups of respondents with one another and to compare rates with historical data. No missing data imputation procedures were employed as missing data are a crucial feature when determining data completeness. Rates of missing data will be presented here.

If a patient answered one question from the first column of every page (i.e. set of questions like “have you been/seen/taken…”) with “yes” and left out all the others items, we counted the left out answers as a “no”.

## Results

### Translation and pretest

During the translation process it became clear that adaptations were necessary to ensure the questionnaire was comprehensible to users of the German health care system, e.g. there is no herbalist in the German system, but a medical specialist for CAM (Arzt für Naturheilverfahren) and a non-medical CAM specialist (Heilpraktiker). Finally, we also had to change the format from upright to the landscape format after doing the pretests. Respondents reported also having difficulties filling in the number of times they had used a particular treatment during the previous 3 months, especially on page 4 where techniques that are generally used regularly are asked about (yoga, relaxation).

### Cognitive interviews

It was easy for those patients who either know a great deal about CAM (N=2), or do not use CAM at all (N=2), to answer the questions. However, it was difficult for those who had only little experience of using CAM therapies because they often did not know the difference between different therapies, how to distinguish one practitioner from another, and how to differentiate between remedies and supplements. Even the difference between a non-medical and medical provider was not always clear to patients. The cognitive interviews resulted in our differentiating between osteopathy, chiropractic and manual therapies. We had first thought these should be lumped together into one item because physiotherapists (medical therapists, but non-MDs) employ all three treatments in Germany. At this stage the “spiritual healer”, “spiritual healing” and “attending a traditional healing ceremony” were scrapped, as was the item (newly introduced for Germany) “anthroposophic remedies” and “anthroposophic medicine”, as respondents had problems with these items. They were either confused by them (mostly spiritual healing), or had no idea how to distinguish between anthroposophic remedies, and homeopathic and herbal medicine.

Providing the main reason for using CAM proved to be challenging for respondents: they either felt that acute symptoms or chronic conditions could not be differentiated from “well-being”, or gave the main reason for visiting the health care provider, regardless of whether the visit was associated with CAM usage.

Manual therapies are difficult to differentiate from each other: there were a lot of questions like “what does a chiropractor do”, “does osteopathy classify as chiropractice”, “do you mean manual therapy given by a physiotherapist or by a chiropractor”, etc.

On page 3, where respondents are asked to fill in brand names of remedies and dietary supplements, they were often unable to remember the correct name, or even the main ingredient of a remedy. Even if they could remember the name of the product, it was often difficult for them to decide to which group the remedy belonged.

Regarding the “number of times” they had sought CAM therapy, it was difficult for respondents to give a specific number whenever it was not easy to separate usage into discrete entities (taking remedies daily over a period of two weeks, taking Yoga classes twice a week, praying regularly several times a day, etc.). All interviewees preferred the newly introduced response option on page 4 “daily, weekly, monthly, less than once a month”, but we also had to add “not at all”.

### CAM use

#### Breast cancer sample

**Figure 2:**
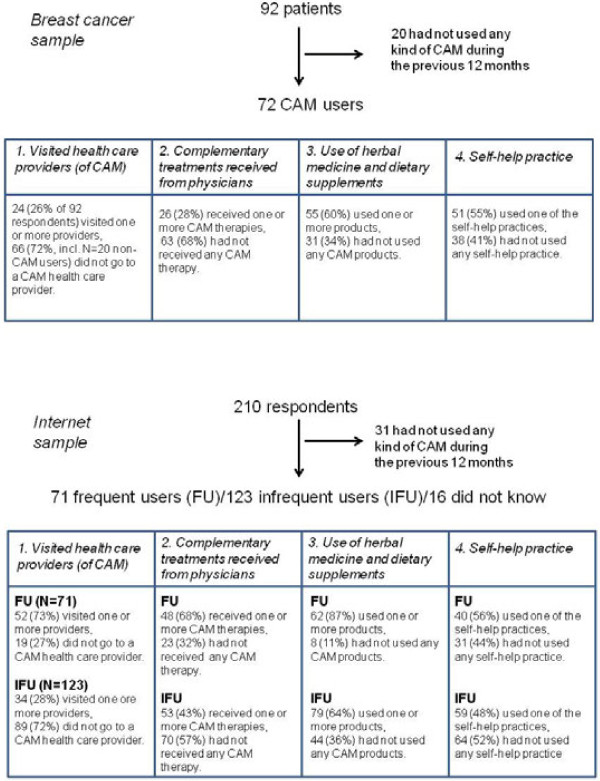
Flow chart for samples.

Ninety-two breast cancer patients filled out the questionnaire. 20 patients did not use any form of CAM. 72 answered at least one question in the questionnaire with yes (please see Figure 2).

1. Visiting health care providers:

26% (N=24) of the 92 respondents had visited at least one CAM practitioner. The highest use was given for medical CAM specialists (Arzt für Naturheilverfahren, 11%) and for non-medical CAM practitioners (Heilpraktiker, 7%; please see Table 2). Number of times the providers were seen during the previous 3 months ranged from 1–24.

**Table 2:** Rates of CAM usage and subjective helpfulness

	***Breast cancer sample (N=72)***			***Internet sample***					
				**Frequent users (N=71)**			**Infrequent users (N=123)**		
**CAM modality**	**use (N,%)**	No of times (valid N, range*)	**How helpful?+ valid N; mean (range)**	**use (N,%)**	**No of times (valid N, range)**	**How helpful?+ valid N; mean (range)**	**use (N,%)**	**No of times (valid N, range)**	**How helpful?+ valid N; mean (range)**
**Visiting health care providers (page 1)**									
Medical CAM specialist	10 (11%)	7 (1–24)	10; 1.4 (1–3)	19 (31%)	8 (0–9)	19; 1.42 (1–3)	11 (9%)	7 (0–8)	10; 1.1 (1–2)
Non-medical CAM practitioner	6 (7%)	4 (1–14)	4; 1.0 (1)	22 (34%)	13 (0–5)	21; 1.05 (1–2)	14 (12%)	10 (0–5)	13; 1.46 (1–2)
Chirotherapist	4 (4%)	3 (0–4)	4; 1.25 (1–2)	5 (8%)	5 (0–4)	5; 1.0 (1)	6 (5%)	3 (0–4)	6; 1.5 (1–2)
Homeopath	3 (3%)	1 (2)	2; 1.0 (1)	26 (41%)	16 (0–7)	25; 1.24 (1–3)	3 (2%)	3 (1–4)	2; 2.0 (1–3)
**Complementary therapies received from physicians (MDs) (page2)**									
Herbs	21 (23%)	10 (1–14)	15; 1.53 (1–3)	30 (46%)	27 (0–40)	27; 1.33 (1–3)	44 (36%)	41 (0–10)	40; 1.63 (1–3)
Acupuncture	6 (7%)	1 (10)	2; 1.5 (1–2)	10 (15%)	8 (0–10)	8; 1.25 (1–3)	4 (3%)	4 (0–20)	4; 1.25 (1–2)
Homeopathy	5 (5%)	4 (2–14)	6; 1.33 (1–2)	23 (34%)	20 (0–270)	23; 1.39 (1–3)	6 (5%)	6 (0–1)	4; 1.5 (1–2)
**Use of herbal medicines and dietary supplements (page 3)**									
Homeopathic remedies	16 (17%)	14 (10 currently**)	13; 1.23 (1–3)	36 (53%)	33 (12 currently**)	33; 1.33 (1–3)	27 (24%)	26 (11 currently**)	23; 1.3 (1–2)
Herbs/ Herbal medicines	30 (33%)	26 (22 currently**)	19; 1.37 (1–3)	38 (58%)	34 (11 currently**)	35; 1.29 (1–2)	40 (35%)	40 (13 currently**	37; 1.54 (1–3)
Vitamins	37 (40%)	31 (27 currently**)	22; 1.23 (1–2)	34 (50%)	32 (23 currently**)	27; 1.41 (1–3)	46 (38%)	46 (29 currently**)	42; 1.48 (1–3)
**Self-help practices (page 4)**									
Meditation	15 (16%)	12 (daily-not at all)	14; 1.43 (1–2)	16 (25%)	16 (daily-less than once a month)	15; 1.07 (1–2)	13 (11%)	13 (daily, weekly-less than once a month)	12; 1.25 (1–2)
Yoga	8 (9%)	6 (daily-monthly)	7; 1.57 (1–2)	10 (16%)	10 (weekly-not at all)	9; 1.11 (1–2)	18 (16%)	18 (daily-not at all)	18; 1.39 (1–2)
Qigong	4 (4%)	4 (daily -not at all)	4; 1.5 (1–2)	10 (16%)	10 (weekly-not at all)	9; 1.11 (1–2)	18 (16%)	18 (daily-not at all)	18; 1.39 (1–2)
Tai Chi	4 (4%)	4 (daily- not at all)	3; 1.67 (1–3)	1 (2%)	1 (weekly)	1; 1.0 (1)	4 (4%)	4 (weekly)	4; 1.25 (1–2)
Relaxation techniques	18 (20%)	14 (daily-not at all)	15; 1.33 (1–2)	24 (35%)	24 (daily-less than once a month)	21; 1.29 (1–3)	31 (26%)	31 (daily-not at all)	30; 1.53 (1–3)
Visualization	7 (8%)	7 (daily-less than once a month)	7; 1.0 (1)	12 (18%)	12 (daily-less than once a month)	11; 1.18 (1–2)	10 (8%)	10 (daily-less than once a month)	9; 1.33 (1–2)

* please see response options for page 3 and 4 in text.

**Refers to the first remedy provided in questionnaire.

+ rating scale ranging from 1 (very helpful) to 3 (not helpful at all), valid N excludes respondents that ticked “I don’t know”.

2. Complementary treatments received from physicians:

28% (N=26) of the 92 breast cancer patients had been given one or more CAM therapies by a physician, while 68% (N=63) had not received any CAM treatment, or had not visited a physician during the previous 12 months at all (see Figure 2). The highest usage was given for herbal treatment (23%), while others (acupuncture, homeopathy) were substantially lower (<10%) (see Table 2). Number of times the CAM treatments were prescribed or received during the previous 3 months ranged from 1–14.

3. se of herbal medicine and dietary supplements:

60% (N=55) of the 92 breast cancer patients said they had used at least one product, while 34% (N=31) had not used any CAM product at all. In this sample, the highest use was given for vitamins/supplements (40%), but herbal and homeopathic remedies had also been used frequently (see Table 2).

4. Self-help practices:

55% (N=51) patients had used at least one of the self-help practices, and only 41% (N=38) had not used any self-help practices. The highest usage was given for praying (42%), the lowest for Qigong and Tai Chi (4%).

“Number of times you have used this practice during the last 3 months” was changed and the response options “daily, weekly, monthly, less than once a month and not at all” were provided. Most patients used self-help practices like relaxation techniques, meditation and yoga weekly, while practices like visualization and praying were generally used daily.

#### Reasons for using CAM and “helpfulness”

Reasons for seeing a CAM practitioner were most often to improve well-being, but also to treat a long-term illness (see Table 3 for exemplary items). Overall effectiveness was rated rather highly (see Table 2). The response option “I don’t know” (for effectiveness) was used quite often (prescribed herbs 10%; use of herbal medicine 17%, vitamins 22%).

**Table 3:** Reasons for CAM usage (exemplary items per page)

	**Breast cancer sample**					**Internet sample**									
						**Frequent users**					**Infrequent users**				
	**N**	**acute**	**long-term**	**acute + long-term**	**well-being**	**N**	**acute**	**long-term**	**acute + long-term**	**well-being**	**N**	**acute**	**long-term**	**acute + long-term**	**well-being**
Visited a homeopath	**3**	0	2 (67%)	0	2 (67%)	**26**	6 (23%)	13 (50%)	3 (12%)	10 (38%)	**3**	2 (67%)	1 (33%)	0	0
Herbal Medicine	**21**	3 (14%)	8 (38%)	0	13 (62%)	**30**	20 (67%)	3 (10%)	4 (13%)	10 (33%)	**44**	27 (61%)	9 (20%)	2 (5%)	9 (20%)
Vitamins	**37**	7 (19%)	10 (27%)	0	20 (54%)	**34**	5 (15%)	7 (21%)	1 (3%)	21 (62%)	**46**	4 (9%)	7 (15%)	2 (4%)	35 (76%)
Relaxation techniques	**18**	1 (6%)	4 (22%)	0	11 (61%)	**24**	1 (4%)	5 (21%)	1 (4%)	20 (83)	**31**	1 (3%)	6 (19%)	1 (3%)	24 (77%)

#### Internet sample

214 respondents filled in the internet questionnaire. Four (2%) of these did not answer any questions. The sample was split into groups, depending on the answer to the introductory question (“what is your preferred choice when you are ill or feeling unwell?”), 71 (34%) answered “CAM” (referred to as frequent users throughout this article), 123 (59%) answered “conventional medicine” (referred to as infrequent users), and 16 (8%) answered “I don’t know”. For further analysis we left out these 16 data sets as the answer does not distinguish adequately between groups of respondents. Overall, 4 (6%) frequent users and 23 (19%) infrequent users had used no CAM at all during the previous 12 months. Figure 2 shows that 31 respondents of the total sample had not used any CAM at all, meaning that 85% of this sample from the general population had used some form of CAM.

1. Visiting health care providers:

52 (73%) of the 71 frequent users had visited at least one CAM practitioner. In this sample 41% had visited a homeopath, 34% a non-medical CAM specialist and 31% a medical CAM specialist. Number of times the providers were seen during the previous 3 months ranged from 0–9 (please see Table 2).34 (28%) of the 123 infrequent users had visited at least one CAM practitioner. The highest use was given for non-medical CAM specialists (12 %). Number of times the providers were seen during the previous 3 months ranged from 0–8.

2. Complementary treatments received from physicians:

48 (68%) frequent users had received at least one CAM therapy from a physician. The most commonly used CAM methods were herbs (46%), followed by homeopathy (34%) and acupuncture (15%). Number of times the CAM treatments were prescribed or used during the previous 3 months ranged from 0–270 (homeopathy).

53 (43%) infrequent users had received at least one CAM therapy from a physician. Herbs were the most common choice (36%). Number of times the CAM treatments were prescribed or used during the previous 3 months ranged from 0–20.

3. Use of Herbal Medicine and Dietary Supplements:

62 (87%) frequent users had used at least one CAM product, while 79 (64%) infrequent users had used at least one of these remedies.

About 58% of frequent users used herbs, 53% homeopathic remedies and 50% vitamins/minerals. The highest rates for use of remedies and dietary supplements in the infrequent users group were given for vitamins/minerals (38%) and herbs (35%).

Infrequent users tended to prefer compound remedies to the pure herbal or classical homeopathic remedies favored by the frequent users.

4. Self-help practices

40 (56%) frequent users practiced at least one self-help technique, while 59 (48%) infrequent users did so.

In the frequent users group, 35% used relaxation techniques and 25% did meditation. In the infrequent users sample the most commonly used self-help practice were relaxation techniques (26%).

#### Reasons for using CAM and “helpfulness”

Reasons for seeing a CAM practitioner were most often to improve well-being, but also to treat a long-term illness (see Table 3). The helpfulness of CAM treatments was rated rather highly overall (see Table 2). Among practitioners, frequent users rated seeing a CAM medical specialist as least helpful, while infrequent users reckoned seeing a homeopath was least helpful.

#### Measurement properties

Only a few respondents failed to fill in entire pages (data not shown), but the amount of missing data was highest for the numbers of times practitioners were seen (the number of valid answers can be found in Table 2). Interestingly, respondents found it easier to rate helpfulness than to provide details on the number of times an event had occurred, thus the item “helpfulness” has higher rates of valid N than the item “number of times”. The most important aspects of missing data per section are:

Section 1: visiting health care providers

About 2/3 of the respondents from the breast cancer sample who ticked a CAM modality also filled in the item “No. of times” (please see valid N in 3rd column of Table 2). Missing data for filling in “number of times” in the internet sample was up to 58% for seeing a Medical CAM Specialist and was also high (around 40%) for the other items in both groups.

Section 2: Complementary treatments received from physicians

Missing data in response to the question “No. of times” ranged from 20% to 83% in the breast cancer sample, and was much lower in the internet sample.

Section 3: Use of Herbal Medicine and Dietary Supplements

Knowledge of herbal remedies (80%) and vitamins (89%) was quite good in the breast cancer sample (brand name referred to the correct category, data not shown), whereas only 56% of homeopathic remedies genuinely belonged to the category of homeopathic remedy (others were complex remedies, fruit essences, or herbal formulas like Echinacea or mistletoe). 10 products were placed in “other CAM remedies”. These were spices, minerals, herbs, complex remedies and tea formulae. Missing data referring to current use totalled only 20% in this sample.

Missing data for the number of times remedies had been used totalled less than 10% in the internet sample.

Knowledge of Vitamins and Minerals (82%) was fairly good in the frequent users group, whereas only 75% of homeopathic remedies and 66% of herbs were assigned to the right category. Among homeopathic remedies, most wrong placements were herbs and among herbs most wrong placements were homeopathic remedies.

Knowledge of vitamins and minerals in the infrequent users group was also quite good (92% of products were placed in the correct category). Knowledge of homeopathic remedies (77%) and herbs (74%) was a little lower.

Section 4: Self-Help-Practices

Missing data was highest for giving the number of times for “Praying for own health” (39%) in the breast cancer sample. It is of note that “No of times” was changed to “daily” and “less than once a month” in all samples, and these answers were filled in by nearly all patients from the internet sample (92-100%).

## Discussion

We conclude from our data that the questionnaire appropriately measures CAM treatment options sought or received during the previous year, provided that some cultural adaptations are made to the English version.

As missing data may threaten validity, it is worth looking at rates of missing responses very closely to assess measurement properties of a questionnaire. It can be concluded that the rate of missing responses was acceptable for overall CAM usage within the previous 12 months, and for ratings of helpfulness. Information on the number of times a certain CAM modality had been used during the previous 3 months was rather limited, as missing data rates were as high as 50% for these items. Due to its format, the paper-pencil-version of the questionnaire yielded more missing data than the internet version. The reason for choosing a CAM treatment option was not filled in according to the pre-defined response options as respondents did not differentiate between acute and chronic conditions, and improving their general well-being. Non-CAM users tended not to fill in the I-CAM-G in the paper-pencil version, and returned a blank questionnaire saying that “this” does not apply to them.

### Possible Bias

The steps undertaken in this study can be considered to represent a comprehensive adaptation of an English questionnaire and the study gives results for different groups of possible respondents as well as assesses missing data. However, this is by no means a full validation study, but rather looks at the performance of the instrument in these groups. The groups are chosen in accordance with other studies [4-7] and with ethnomedical traditions in Germany, but clearly could be seen as somewhat arbitrary. We employed the questionnaire both in paper-pencil-format and as an internet survey in order to get some impressions about which form might be suitable, but as we did not employ both forms in all samples this can be seen only as exemplary.

### Comparison with other studies

We looked at the performance of the ICAM-Q by comparing results with historical data, as was done in another recently published study [17]. Our study adds to this comparison some measurement properties like meaning of items and missing data.

In our study the overall results for CAM usage in the breast cancer sample resemble those reported in other studies in cancer patients in German-speaking countries like Switzerland and Europe [18]. However, the impression of underestimating use with the ICAM-Q [17] can be seen as partly supported by our results as the use of breast cancer patients in a European study exceeds the use in our study, especially with respect to homeopathy and herbal medicine [19].

There was significant misplacing of herbal and homeopathic remedies in our study, when respondents were asked to put them in pre-specified categories. This is exactly in line with other findings, which showed that misinterpretation are likely as respondents do base their ratings on established definitions, and even incorporate notions of self-concept [17].

### Implications

By means of a methodologically sound translation and an adaptation process, we were able to transform a published international questionnaire for measuring CAM use (I-CAM-Q) into a German version of the measurement instrument (I-CAM-G). The I-CAM-G is comparable with the international (English) measurement instrument and is also suitable for use within the German health care system. Adaptions to the English version proved to be necessary, and these adaptations must take the policy situation in a specific country into account (e.g. reimbursement scheme of non-medical providers) and ethnomedical traditions.

All in all, CAM use could appropriately be measured using the I-CAM-G, and other research shows that prompting is the best method to elicitate practices used [20].

However, we would recommend developing a short version of the questionnaire which nevertheless retains the basic structure of the original (4 parts asking about CAM treatments, providers seen, particular remedies, self-help practices), but does not ask for details on the number of times during the previous 3 months and reasons for use. The items referring to “number of times during the last 3 months” bear the risk of remaining unanswered, thus resulting in missing data, and the reason for seeking CAM could then not be reliably answered. The short version should include items related to subjectively perceived helpfulness, and might skip the CAM modality under which a particular remedy has to be placed, but instead ask for a list of remedies. In case one would be interested in measuring CAM treatments in order to compare use of CAM options to use of medical non-CAM options one might consider leaving out the self-help practices. This short version would allow to feasibly measure important issues like visiting CAM providers, use of complementary therapies and remedies and perceived helpfulness of these options.

## Conclusions

The I-CAM-G is suitable for measuring different kind of CAM therapies and techniques and their perceived helpfulness. More detailed information on number of times and reasons for use could not be adequately measured, at least not when respondents are asked to fill in the paper-pencil-questionnaire or an internet survey by themselves.

## Competing interests

The authors declare that they have no competing interests.

## Authors’ contributions

CG designed the study, carried out the analysis, and drafted the manuscript. MLR designed the questionnaire graphically, carried out the interviews and the questionnaire studies, and helped to draft the manuscript. SS provided psychometric support and helped to draft the manuscript. All authors read and approved the final manuscript.

## Pre-publication history

The pre-publication history for this paper can be accessed here:

http://www.biomedcentral.com/1472-6882/12/259/prepub
